# Metabolomics Research Reveals the Mechanism of Action of Astragalus Polysaccharide in Rats with Digestive System Disorders

**DOI:** 10.3390/molecules23123333

**Published:** 2018-12-15

**Authors:** Huanjun Wang, Ana Liu, Wenxiao Zhao, Haijun Zhao, Lili Gong, Erdong Chen, Ning Cui, Xuming Ji, Shijun Wang, Haiqiang Jiang

**Affiliations:** 1 School of Pharmaceutical Sciences, Shandong University of Traditional Chinese Medicine, Jinan 250355, China; zyywhj123@163.com (H.W.); lwtgyx@hotmail.com (A.L.); ced777@163.com (E.C.); 2 College of Nursing, Shandong University of Traditional Chinese Medicine, Jinan 250355, China; Zhaowx912@163.com; 3 College of traditional Chinese medicine, Shandong University of Traditional Chinese Medicine, Jinan 250355, China; higherzhao@126.com (H.Z.); cuining_sdutcm@163.com (N.C.); jixuming724@163.com (X.J.); 4 Experiment Center of Shandong University of Traditional Chinese Medicine, Jinan 250355, China; Liligong@sdutcm.edu.cn

**Keywords:** Astragalus polysaccharide, digestive system disorders, metabolomics

## Abstract

With the diversity of modern dietary lifestyles, digestive system disorders (DSD) have become a frequently occurring disease in recent years. Astragalus polysaccharide (APS) is a homogeneous polysaccharide extracted from *Astragalus*, which might ameliorate the digestive and absorptive functions. However, the treatment mechanisms remain unclear. In this study, rats with DSD were fed a high-fat–low-protein diet and subjected to weight-bearing swimming until exhaustion. When body weight and autonomous activities of the rats decreased, they were administered APS. After two weeks, serum metabolomics analysis based on LC-MS was performed to validate the therapeutic effect of APS and explore its mechanism. APS pharmacodynamics was determined in this study, and serum metabolomics analysis discovered and identified 16 significant, differentially produced metabolites involved in energy, amino acid, and lipid metabolism, including citric acid, lactic acid, alanine, phosphatidylcholine, phenylalanine. After treatment with APS, the levels of the above small-molecule metabolites were reversed. Our results show the efficacy of APS in DSD treatment through the regulation of perturbed metabolic pathways related to energy, amino acid, and lipid metabolism.

## 1. Introduction

Digestive system disorders (DSD) have become a widespread disease, and the prevalence of DSD has been increasing owing to irregular dietary habits and contaminated food. DSD greatly reduce life quality. The most important consequence of DSD is compromised nutrient absorption [1]. DSD can increase gastric sensitivity to distension and delay gut transit. [2]. Stomach fullness, loss of appetite, inability to finish a meal, excessive postprandial fullness, bloating, nausea, retching, and vomiting are the main symptoms of DSD [3], which might favor the development of other diseases in the long run, including liver cirrhosis [2], mental disorders [4], etc. Epidemiological statistics displayed that as of 2012, gastrointestinal diseases were an important source of substantial morbidity and mortality and caused the highest healt expenditures in the United States [5]. DSD has bloomed into a burden in some areas, such as India [6] and Portugal [7]. It is urgent to find effective drugs to cure DSD.

Astragalus polysaccharide (APS) is a homogeneous polysaccharide extracted from Radix Astragali (root of Radix Astragali (Fisch.) Bge. var. mongholicus (Bge.) Hsiao or Radix Astragali (Fisch.) Bge.). Several studies have indicated that APS has a wide range of therapeutic effects, including ameliorating chemotherapy-induced cardiotoxicity [8] and ionizing radiation-induced oxidative stress [9], regulating inflammatory reactions [10], treatment of diabetes [11], as well as antitumor activity and enhancement of the immune function [12]. DSD includes gastrointestinal dysfunction. It was reported that APS could significantly improve the colitis disease activity index (DAI) and histological scores, as well as increase weight and colon length in mice [1]. APS could also decrease the production of inflammatory factors such as TNF-α, IL-1β, IL-6, IL-17 [13,14] in a study in a mouse model of dextran sulfate sodium (DSS)-induced colitis. In addition, a study on an APS-based intervention for chronic atrophic (CAG) induced by N-methyl-N’-nitro-N-nitrosoguanidine (MNNG) in rats found that the gastric morphology improved, and plasma gastrin and somatostatin levels significantly increased [15]. These studies indicate that APS has a good effect on DSD. Yet, reports on the changes of the serum levels of small molecules in DSD-affected rats are rare. A recent study has demonstrated that dietary supplementation with APS enhanced ileal digestive and absorption function through regulating amino acids metabolism [16]. Although this study is focused on the regulatory effect of APS on amino acids, it also briefly presents the effects on other endogenous metabolites, which, nevertheless, was not conducive to explaining the mechanism of APS in the treatment of the DSD. In order to explore the mechanism of small molecule changes after APS treatment of DSD from the microscopic and holistic perspectives and to show the material and theoretical basis for clinical medication, we chose to perform serum metabolomics to determine the changes in small molecule metabolites from a holistic perspective. According to clinical studies, patients with digestive system dysfunction generally lose weight and reduce the level of independent activities. Therefore, body weight and spontaneous activity were recorded to verify if the model was valid and to explore the therapeutic effect of APS. In this study, metabolomics analysis was helpful to shed light on the mechanism of APS in treating DSD and to clarify the Chinese medicine ingredients effective in the treatment of diseases. 

Metabolomics is a powerful tool to analyze the changes of endogenous small molecules in the organism. Compared to other “omics”, it has the advantage to directly indicate changes in gene transcription and protein levels, thus allowing to characterize specific metabolic phenotypes [17]. Therefore, metabolomics is commonly used to study homeostatic regulation and system perturbations [18]. DSD lead to alterations of many small molecules in the body. In view of this, understanding the changes of small molecules in the body on the basis of metabolomics is essential to clarify the mechanisms of development of diseases and thus design prevention strategies. Therefore, this study will help to get a better understanding of the protection mechanism of APS against DSD, based on untargeted LC-MS.

## 2. Results 

### 2.1. Animal Model of DSD

**Figure 1:**
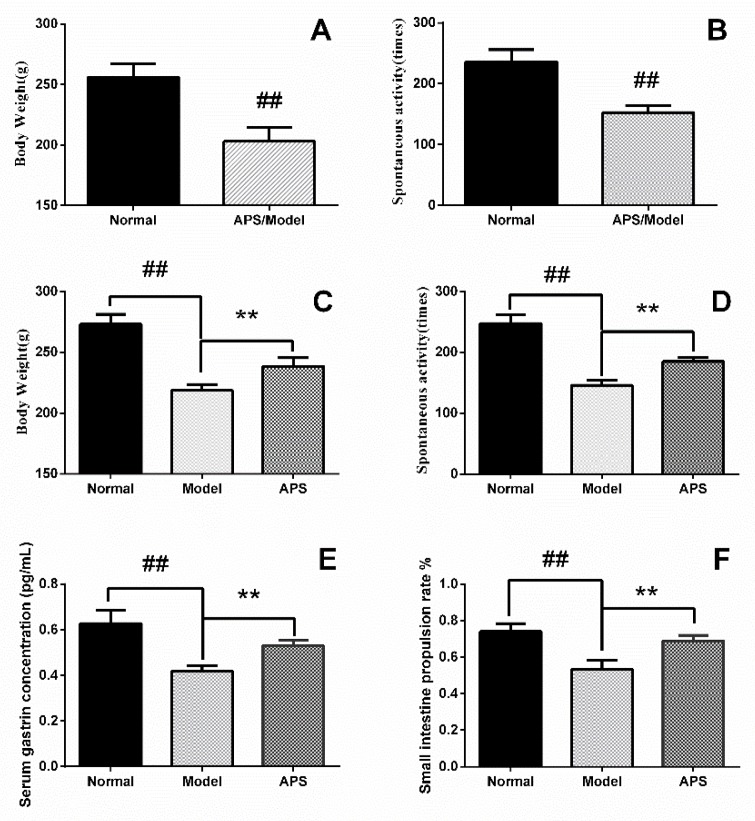
Body weight and spontaneous activity of the control (normal) group and the Astragalus polysaccharide (APS)/model group (**A**), (**B**). The values given are the means ± SD (n = 7 in normal group, n = 14 in APS/model group). After APS intervention, the change of body weight, spontaneous activity, gastrin concentration, and small intestine propulsive rate in the normal group, model group, and APS group are shown in (**C**), (**D**), (**E**), (**F**). The values given are the means ± SD (n = 7); ^##^ *p* < 0.01 compared with the normal group, ** *p* < 0.01 compared with the APS group.

During the establishment of a DSD model, rats were divided into a control group of normal rats (n = 7), and an APS/model group (n = 14). During the first week of modeling, the amount of food given to rats in the APS/model group decreased, and the weight increased slowly. After two weeks, rats’ spontaneous activity was reduced, and squinting signs appeared. After four weeks, the rats had appeared arched, and the color of their fur became dark. After six weeks, the ingested food and body weight of the rats in the APS/model group drastically decreased, which was accompanied by mental sluggishness. The last measured body weight and spontaneous activity of the APS/model group are shown in Figure 1A and Figure 1B, respectively. We used the same method as in a previous study to establish a digestive system disorder model. The symptoms we observed were quite consistent with those of the previous experiments, such as weight loss, reduced autonomic activity, etc. [19]. This means that a DSD animal model has been successfully established [20]. Then rats in the APS/model group were divided into an APS group (n = 7) and a model group (n = 7). To demonstrate the therapeutic effect of APS, we determined body weight, spontaneous activity, gastrin concentration, and small intestine propulsive rate in the rats. The results showed these indicators increased significantly after treatment with APS, as shown in Figure 1C–F, respectively, indicating that APS has a good therapeutic effect on digestive tract disorders. 

### 2.2. Multivariate Statistical Analysis of Metabolomics Data

**Figure 2:**
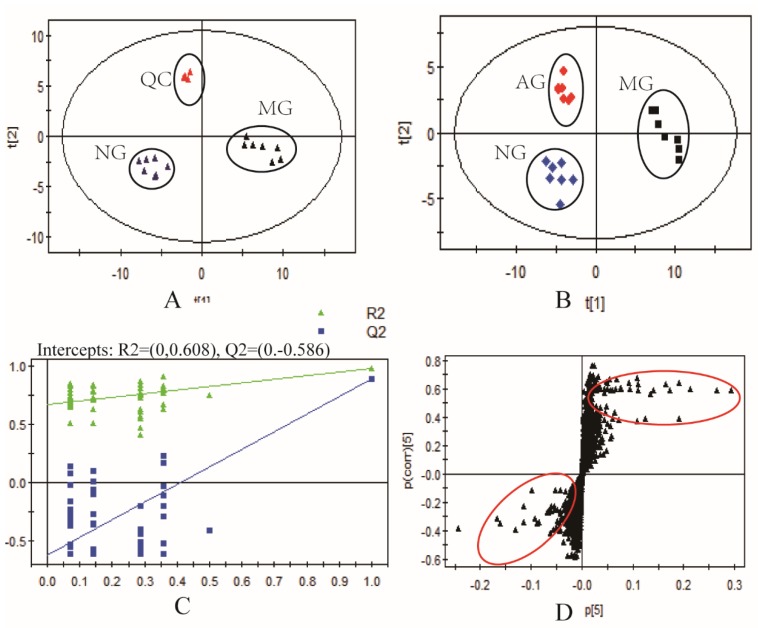
Results of multivariate statistical analysis of the serum data. (**A**): Principal component analysis (PCA) score plot of the normal group, model group, and quality control (QC) samples. (**B**): partial least-squares-discriminant analysis (PLS-DA) score plot based on the LC-MS data for the normal group, model group, and APS-treated group. (**C**): validated model plots based on the LC-MS data. (**D**): S-Plot of PLS-DA based on the serum metabolic data.

The mass spectrometry data of quality control (QC) samples acquired from LC-MS were pretreated with XCMS software, and the result of principal component analysis (PCA) score plot are shown in Figure 2A. All QC samples were clustered together and concentrated in the 95% confidence interval, suggesting the instrument was working properly, and the data quality was reliable. In order to search the differences and relationships among the three groups, multivariate statistical analysis methods were applied, including PCA and partial least-squares-discriminant analysis (PLS-DA). The results of PCA are shown in Figure 2A. Our results showed that the normal group and the model group were effectively separated, and the cohesion in the same group was fine. The results implied that the animal model was established successfully, and the serum metabolic patterns of the model group and the normal group were different. PLS-DA was applied to discover the contributable variable among the three groups (Figure 2B). In the PLS-DA model, cumulative R2Y and Q2 were 0.981 and 0.912, respectively. (R2Y is the fitting ability of the model, Q2 is the prediction ability of the model, high values of both mean a successful model), while the intercepts of R2 and Q2 were 0.608 and 0.586 (100 random arrangement tests, Figure 2C). These data suggested that the PLS-DA models were reliable and valid. An S-Plot was acquired to screen for biomarkers (Figure 2D). S-plots can reflect the covariance and correlation between a model and the variables. The farther away from the origin are the spots, the greater the classification ability, which may lead to the identification of potential biomarkers. S-plots can reduce the false positive rate in potential marker selection.

### 2.3. Identification of Potential Biomarkers

**Figure 3:**
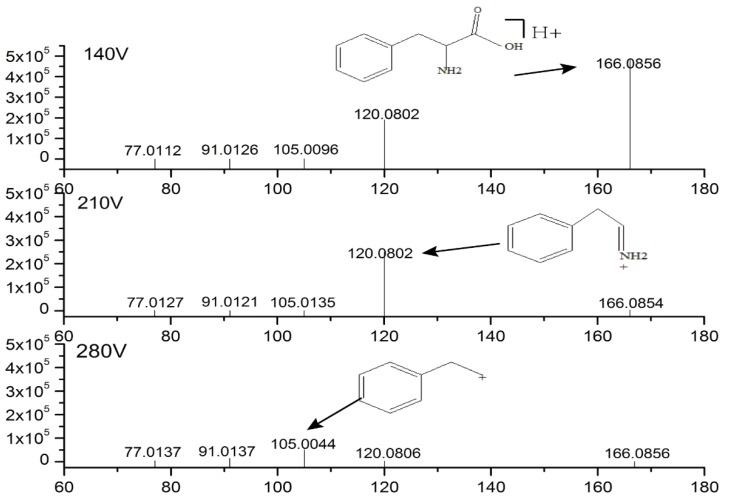
Analysis of phenylalanine structure.

The metabolites with variable importance projection (VIP) > 1, *p* < 0.05, fold change (FC) ≥ 2 were selected as potential biomarkers. The exact mass of the selected as metabolites were retrieved in HMDB (http://www.hmdb.ca), METLIN (https://metlin.scripps.edu), and KEGG (http://www.genome.jp/kegg/) databases and combined with mass spectrometry fragmentation information to confirm the compounds structure. As an example, phenylalanine was chosen to illustrate the process of structure identification. The exact mass of phenylalanine is 165.079, and its characteristic ion pair is (166/120). According to the retention time, we could confirm that the 166.0853 *m*/*z* signal at 140 V fragmentation voltage was the [M + H]^+^ peak of phenylalanine, and the 166.0853 *m*/*z* signal showed the same retention time at 210v and 280v fragmentation voltage (however, most fragments were destroyed, so the 166.0853 *m/z* signal at 210v and 280v fragmentation voltage showed a lower response). At 210 V fragmentation voltage, the 120.0799 *m/z* signal corresponded to the characteristic ion of phenylalanine derived from the loss of the –COOH fragment from phenylalanine. At 280 V fragmentation voltage, the 105.0441 *m/z* signal was derived from the loss of the –NH2 group from the fragment corresponding to the 120.0799 *m*/*z* signal. On the basis of the above information, we determined that the 166.0853 *m/z* signal corresponded to phenylalanine. Mass spectrometric data information and results are displayed in Figure 3. Finally, 16 different biomarkers were identified (Table 1).

### 2.4. Heat Map Analysis

**Figure 4:**
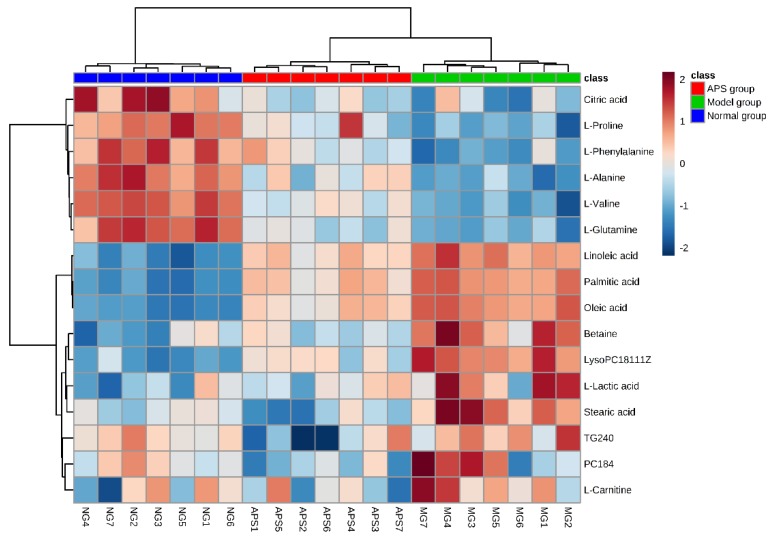
Heat map of the 16 differentially produced endogenous metabolites between the normal group, model group, and APS group. Orange plots indicate up-regulated metabolites, and blue plots indicate down-regulated metabolites in rats.

**Figure 5:**
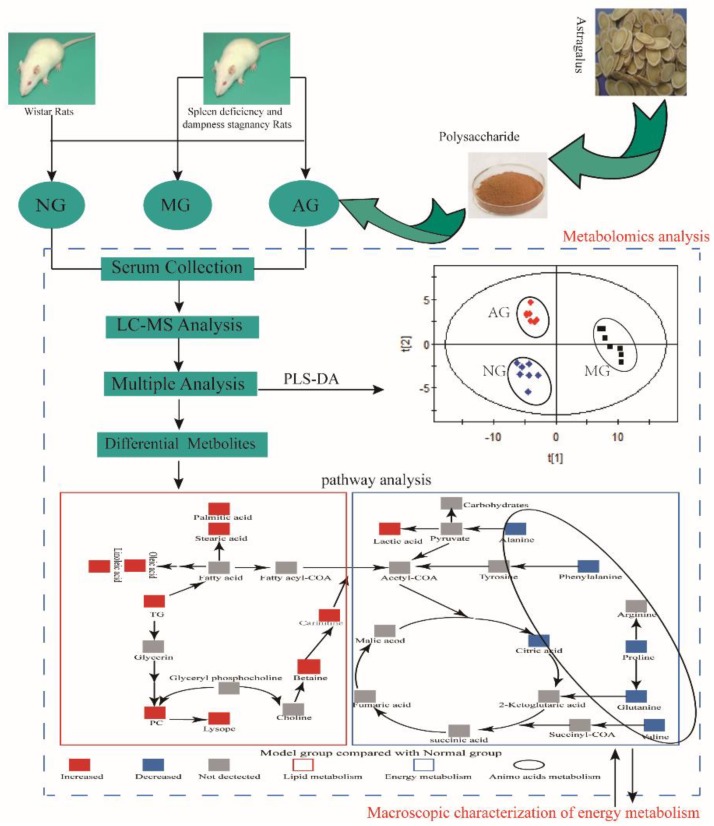
Experimental flow chart of the overall behavioral and serum metabolomic approaches.

The relative amounts of metabolites in the three groups were clearly visualized by a heat map, and hierarchical cluster was analyzed, as shown in Figure 4. Our results showed that the levels of 10 metabolites including phosphatidylcholine (PC), lysophosphatidylcholine (LysoPC), palmitic acid, stearic acid, linoleic acid, oleic acid, lactic acid, carnitine, betaine, and triglycerides (TG, *p* < 0.01) were higher in the model group compared with the normal group, whereas the levels of alanine, valine, glutamine, citric acid, proline, and phenylalanine were all decreased (*p* < 0.01). After intervention with APS, the content of 16 metabolites returned to the normal level. All of the 16 biomarkers were involved in linoleic acid metabolism, phenylalanine, tyrosine, and tryptophan biosynthesis, phenylalanine metabolism, valine, leucine, and isoleucine biosynthesis, glyoxylate and dicarboxylate metabolism, alanine, aspartate, and glutamate metabolism, glycerophospholipid metabolism, arginine and proline metabolism, citrate cycle (TCA cycle). On the basis of metabolic pathways and metabolites, we also produced an experimental network map, so as to clearly interpret the results obtained (Figure 5). 

## 3. Discussion

In our experiment, body weight, spontaneous activity, gastrin concentration, and small intestinal propulsion rate of rats in the normal group, model group, and APS group appeared different. These variables indicated the disordered digestive system function of the rats in the model group. DSD can result in dysfunctions during transformation and transport of nutritive molecules and in inadequate intake of nutrients, which leads to insufficient energy metabolism. Moreover, the rats of the model group displayed a series of symptoms such as loss of weight, reduction of autonomic activity, etc. After the treatment with APS, the function of the intestine and stomach was restored. Our study provides novel evidence to further explore the mechanism of APS in the treatment of DSD.

### 3.1. Energy Metabolism 

From the molecular point of view, the levels of metabolites related to energy metabolism, such as citric acid and lactic acid, were significantly different, when comparing the metabolites of the model group with those of the normal group. Citric acid is an important intermediate in the citric acid cycle, which takes place in the mitochondria [21]. The citric acid cycle is the hub of glycometabolism, and lipid and amino acid metabolism and provides the reduced form of nicotinamide-adenine dinucleotide (NADH), a proton (H^+^), and flavin adenine dinucleotide, generating ATP to supply energy for the body, through electron transfer and oxidation. Citric acid reduction in the model group indicated a disorder of the citric acid cycle and insufficient energy supply [22], leading to mental sluggishness in the rats. Lactic acid plays an important role in healing, ischemic tissue injury, and cancer growth and metastasis. Lactic acid can accumulate in a number of pathological conditions [23]. In energy metabolism, lactic acid diminishes cellular cAMP, resulting in a reduction of lipolysis and eventually conserving fats [24,25,26]. This partly explains why TG (24:0) increased in the rats of the model group. However, after intervention with APS, the changes in the levels of citric acid and lactic acid in the treated rats meant that the disease condition was improved, and the digestive system function was gradually restored.

### 3.2. Amino Acid Metabolism

Phenylalanine is an essential amino acid and a glycogenic amino acid. It can be transformed into dopamine, epinephrine, and tyrosine in vivo [27]. Tyrosine continues to decompose to produce acetoacetic acid and fumaric acid, and the latter participates in the TCA cycle to provide energy for the body. Valine, one of the components of the branched chain amino acids (BCAA), is a dietary indispensable amino acid [28]. Succinyl-CoA or acetyl-CoA are the end product of valine and can enter the TCA cycle to generate energy and gluconeogenesis or act as precursors for lipogenesis and ketone body production through acetyl-CoA and acetoacetate [29]. The exhaustive swimming method, which is equivalent to strenuous activity, was utilized to establish the model. It appears that phenylalanine and valine are required to accelerate metabolism and maintain body homeostasis. Therefore, the decreased levels of phenylalanine and valine in the body are consistent with this mechanism. However, after administration of APS, the levels of phenylalanine and valine increased. It has been reported an inverse association between BCAA and adipokines in diabetes [30,31]. The results of our study on DSD are consistent with these reports: under the influence of a high-fat and low-protein diet plus exhaustive swimming, glycemic amino acid decreased, while triglycerides for energy supply and fatty acids increased. In addition, we also found that betaine increased in the model group. Betaine is distributed widely in animals and plants, and its principal physiologic roles are to regulate osmotic pressure, protect internal organs, decrease vascular risk factors, and enhance performance [32]. Gastrointestinal dysfunction in rats with DSD hinders the regular nutrients delivery to all parts of the body. We assumed that the osmotic pressure was increased. In order to ensure a relative homeostasis, betaine content increases to maintain osmotic pressure stability, which is consistent with our experimental results.

### 3.3. Lipid Metabolism

Disturbances in fatty acid metabolism play an important role in the development of impaired glucose metabolism [33]. When energy is required (e.g., during overtraining in our experiment), TG, stored in intracellular lipid droplets, are hydrolyzed via the activation of the intracellular lipolytic pathway to release fatty acids [34], However, due to the high fat content in the diet of the rats in the model group, the serum TG (24:0) content in the model group was higher than in the normal group. Under this condition, the model group produced energy mainly through lipid metabolism, which resulted in higher fatty acid levels. In addition, lysoPC and PC were also increased. PC can be catalyzed by different types of specific phospholipase (PLA) enzymes to produce lysoPC, which is an important signaling molecule interacting with G protein-coupled receptors in various tissues [35]. It can modulate cellular activities such as cellular permeability, apoptosis, and cell proliferation and migration [36]. However, increased lysoPC and cellular permeability alterations may be responsible for the decrease of body weight in the rats of the model group.

## 4. Materials and Methods

### 4.1. Chemicals and Reagents

Acetonitrile and methanol (HPLC-grade) were obtained from Merck (Darmstadt, Germany). Formic acid (HPLC-grade) was obtained from Fisher Scientific (Loughborough, UK). Ultra-pure water was produced by a Milli-Q Reagent Water System (Millipore, MA, USA). The reference standards of glucose and ethanol (analytical grade) were taken from Sinopharm Chemical Reagent Company (Beijing, China).

### 4.2. Preparation of APS

Radix Astragali was purchased from Shandong University of Traditional Chinese Medicine hospital and authenticated as the dried root of *Astragalus licentianus* of Mongolia by Prof. Zhou Fengqin (School of Pharmacy, Shandong University of Traditional Chinese Medicine). The origin of the herb was in LongXi county, Gan Su province. The crude Radix Astragali was extracted with boiling water for three times (10 times, six times, and six times, respectively), obtaining three extracts. The extracted solutions were filtered and combined, then the water was removed under reduced pressure. Ethanol concentration of the solution was adjusted to 80% then the solution was set aside for one night. The deposition was dissolved in water, and the upper and lower phases were obtained by centrifugation. The ethanol concentration of the upper phase was adjusted to 80%, and the bottom sediment was used as APS. APS was dried to powder under vacuum at 60 °C and finally stored at −20 °C. The phenol-sulfuric acid method was used to determine the content of APS. The content of the APS was 399.1 mg/g; the results of the methodology were within the allowable range. 

### 4.3. Animal Experiment

A total of 21 Wistar rats weighing 150 ± 20 g were purchased from the Experimental Animal Center of Lukang Pharmaceutical Group Co. Ltd. (Jining, Shandong, China). Animal welfare and experimental procedures were strictly in accordance with the Guide for the Care and Use of Laboratory Animals. All animals were housed in the surroundings with a 12 h light–12 h dark cycle, room temperature of 25 ± 1 °C, and relative humidity of 55% ± 5%. Food and water were unrestricted in the experiment. All rats were allowed to acclimatize for one week before the experiment. Then, they were randomly divided into the normal group (n = 7) and the APS/model group (n = 14). The rats of the normal group were fed with a purified diet. The rats in the APS/model group were induced with a high-fat and low-protein diet plus exhaustive swimming to obtain rats with digestive system disorders. After the model was successfully established [14], the APS/model group was randomly divided into the model group (n = 7) and the APS group (n = 7). According to the Chinese Pharmacopoeia (2015 edition), the rats of the APS group were administered APS intragastrically at a dose of 1.41 g per kilogram per day. The rats of the normal group and the model group were administered the same volume of physiological saline. They were all administered by gastric infusion one time each day for two weeks. All animal studies were approved by the Shandong University of Traditional Chinese Medicine animal ethics committee.

### 4.4. Behavior Detection 

The body weight of the rats was measured weekly throughout the study period. Changes in body weight were observed in the three groups; the rats were placed in the independent activity box to record their activities during 10 min. 

### 4.5. Collection of Serum Samples 

Blood was collected from the rats at the end of the experiment and centrifuged at 4000 rpm/min for 15 min. The supernatants were transferred into clean tubes and stored at −80 °C. When the serum samples were used, they were melted at 4 °C.

### 4.6. Serum Gastrin Concentration

An Olympus AU5400 automatic biochemical analyzer (Olympus Optical, Tokyo, Japan) was used to detect serum gastrin concentration. The kit of rat gastrin was supplied by Beijing equation biology company (Beijing, China). The instructions of the manufacturer were followed to detect serum gastrin concentration.

### 4.7. Small Intestinal Propulsion Rate

The small intestines of the rats, from pylorus to the ileocecal region, were extracted and infiltrated with physiological saline. In the absence of traction, the small intestines were placed on a board to measure the distances between pylorus and fore-end of the pigment (the length of the pigment) and pylorus and the ileocecal region (the length of the small intestines). They were used to calculate the small intestine propulsive rate.

### 4.8. Preparation of Serum Samples for Metabolomics

A volume of 300 μL of serum was mixed with 600 μL of methanol, then vortexed 1 min to precipitate the proteins. After centrifuging at 4000 rpm/min for 15 min, the supernatant was filtered through a 0.22 μm membrane filter. 

### 4.9. HPLC–TOF/MS Analysis

HPLC-TOF/MS analysis was performed with a 1260 high-performance liquid chromatography, 6230 time-of-flight mass spectrometer (Agilent Technologies Company, Santa Clara, CA, USA), a UV-2550 ultraviolet spectrophotometer (Shimadzu Corporation, Japan), and an Olympus AU5400 automatic biochemical analyzer (Japan). Chromatographic separation was performed on a Halo-C18 column (2.1 × 100 mm, 2.7 μm, advanced materials technology, USA) with a binary solvent system (solvent A: water with 0.05 % formic acid and solvent B: acetonitrile with 0.05% formic acid). The elution gradient employed in this experiment was: 0~10 min, 90%~70% A; 10~20 min, 70%~40% A; 20~28 min, 40% A; 28~38 min, 40%~20% A; 38~45 min, 20%~0% A. The conditions were: flow rate, 0.30 mL·min^−1^; injection volume, 5 μL; column temperature, 25 °C. 

Mass detection was operated in positive mode with the following optimized conditions: capillary voltage, 4.0 kV; drying gas flow, 11 L/min; gas temperature, 350 °C; nebulizer pressure, 35 psig; fragmentor voltage, 140 V; skimmer voltage, 60 V; *m/z* values of the reference ions, 121.050873 and 922.009798. The *m/z* data were collected for 80–1000 Da. Fragmentor voltages at 210 and 280 V were utilized to search the target fragment ion and identify the structure of the biomarkers. In this study, we only collected the metabolic data in positive mode, because the data in negative mode were not ideal.

A volume of 100 μL of serum from each rat was mixed to serve as a QC sample, which was pretreated as described in “Serum sample preparation”. QC samples were used for monitoring the repeatability of the samples and validating the stability of LC-MS during the analysis. The QC samples were analyzed six times in the beginning and randomly arranged after six unknown serum samples.

### 4.10. Data Handling

Body weight, spontaneous activity, gastrin concentration, and small intestine propulsion rate were expressed by means ± SD, and independent samples t-test was used to compare the two groups. The multi-group comparisons were analyzed by one-way analysis of variance (ANOVA) of SPSS 22.0 (SPSS Inc., Chicago, IL, USA). Values of *p* <0.05 were considered significant. Metabolomics data analysis was as follows.

Before data preprocessing, the raw data were firstly converted to the “mzXML” format by ProteoWizard (http://proteowizard.sourceforge.net/) software. Then, XCMS software (University of Auckland, Auckland, New Zealand, version 3.2.2) was applied for peak identification, peak alignment, peak correction, and retention time correction. Correction parameter settings: ppm =10, bw = 10, snthresh = 20 [37], and other settings were set at default values. Finally, a three-dimensional data matrix which consisted of retention time, mass value, and peak intensity was built. Then, the data matrix ere imported into SIMCA-P (version 11.5, Umetrics, Umea, Sweden) software in “.csv” format for multivariate statistical analysis, PCA, and partial least-squares-discriminant analysis (PLS-DA) for pattern recognition. Variable importance projection (VIP) produced by PLS-DA, ANOVA, and fold change (FC) were applied to discover the contributable variable for classification. Finally, the variables with VIP > 1, *p* <0.05 and FC ≥ 2 were treated as potential biomarkers. The exact mass of the potential biomarkers was searched in databases such as HMDB, METLIN, and KEGG for biomarkers identification. The heat map visualized the relative amounts of the metabolites differentially produced and analyzed their hierarchical cluster; pathways analysis was conducted by Metaboanalyst3.0 (http://www.metaboanalyst.ca). 

## 5. Conclusions 

APS as an important part of Radix Astragali has beneficial effects in the body, and our results suggest it is valuable for treating DSD. According to our expectations, APS showed good therapeutic effects on the induced digestive system disease. From a macroscopic point of view, the condition of the APS group was significantly alleviated compared to the model group. Therefore, we were very interested in exploring the mechanisms of action of APS. In this study, a metabolomics analysis based on LC-MS technology was conducted to investigate the mechanisms of action of APS in the treatment of DSD. We identified 16 significantly different biomarkers, including citric acid, amino acids, fatty acids, and lipids, which suggested that energy metabolism, lipids metabolism, and amino acids metabolism were altered in DSD rats. 

Our study suggests that behavioral analysis combined with serum metabolomics strategies is useful to reveal the comprehensive mechanisms of action of traditional Chinese medicine ingredients in the treatment of diseases. The behavioral study showed that APS had therapeutic effects on the general conditions of the model rats, including body weight, independent activity, etc. The molecular study of metabolomics showed that there were obvious differences in the metabolites between the APS group and the model group. According to our results, 16 significantly changed metabolites were identified, and their levels were observed to be reversed by APS administration. The therapeutic effects of APS might be attributed to the recovery of the energy metabolism. These findings will contribute to understand the treatment effects and mechanisms of APS.

## Figures and Tables

**Table 1:** Potential biomarkers identified in the samples and corresponding metabolic pathways.

No	RT (min)	Exact Mass	Biomarkers	VIP	*p*-Value	Change Trend		FC		Pathway
						M/N	T/M	N/M	T/M	
1	1.126	89.047	L-Alanine	1.250	8.37 × 10^−6^	↓	↑	4.59	2.90	Alanine aspartate and glutamate metabolism
2	1.054	90.031	L-Lactic acid	1.306	9.87 × 10^−5^	↑	↓	287	2.24	Glycolytic pathway
3	1.000	117.079	L-Valine	1.285	2.85 × 10^−10^	↓	↑	3.96	2.39	Glycine leucine isoleucine degradation
4	1.001	117.146	Betaine	1.178	2.03 × 10^−8^	↑	↓	4.27	3.01	Fatty metabolism
5	1.089	146.069	L-Glutamine	1.757	8.74 × 10^−7^	↓	↑	7.13	2.68	Arginine biosynthesis purine metabolism
6	1.297	165.079	L-Phenylalanine	1.825	2.28 × 10^−5^	↓	↑	3.60	2.09	Phenylalanine metabolism
7	1.290	192.123	Citric acid	1.493	0.0058	↓	↑	3.23	2.11	Three-carboxylic acid cycle
8	1.002	115.063	L-Proline	1.629	4.35 × 10^−7^	↓	↑	6.09	2.61	Arginine and proline metabolism
9	16.521	776.034	PC(18:4)	3.905	7.01 × 10^−4^	↑	↓	2.58	2.20	Phospholipid metabolism
10	25.114	521.667	LysoPC (18:1(11Z))	1.101	6.58 × 10^−8^	↑	↓	3.68	2.15	Phospholipid metabolism
11	42.535	256.240	Palmitic acid	1.856	2.09 × 10^−21^	↑	↓	2.37	2.03	Biosynthesis of fatty acid
12	24.900	284.271	Stearic acid	1.774	3.06 × 10^−7^	↑	↓	7.93	3.61	Biosynthesis of fatty acid
13	38.232	280.240	Linoleic acid	1.680	1.94 × 10^−17^	↑	↓	3.14	2.16	Linoleic acid metabolism
14	43.266	282.255	Oleic acid	1.819	6.29 × 10^−19^	↑	↓	8.1	2.45	Biosynthesis of fatty acid
15	14.505	161.105	L-Carnitine	1.291	4.39 × 10^−4^	↑	↓	4.42	3.78	Bile secretion
16	19.094	1017.76	TG(24:0)	1.626	0.0029	↑	↓	2.60	2.07	Fatty metabolism

Note: M/N indicates the model group (M) compared with the normal group (N); (↑): up-regulated; (↓): down-regulated; *p* < 0.01 compared to normal group. T/M indicates the APS-treated group (T) compared with the model group; (↑): up-regulated; (↓): down-regulated; *p* < 0.01 vs. the model group. RT: retention time; VIP: variable importance projection; FC: fold change; PC: phosphatidylcholine; TG: triglycerides.
